# Complex Trauma from Child Abuse and Neglect “*I’m not Sure We’re even All Talking about the Same Thing and We’re Probably Not*”:

**DOI:** 10.1007/s40653-024-00648-z

**Published:** 2024-08-03

**Authors:** Eden Thain, Sarah Cox, Amanda Paton, Sarah Shihata, Leah Bromfield

**Affiliations:** Australian Centre for Child Protection, University of South Australia, Justice & Society, Adelaide (South Australia), Australia

**Keywords:** Child abuse, Neglect, Trauma, Complex trauma, Group interviews

## Abstract

**Rationale:**

Trauma from child abuse and neglect requires specialised assessment and intervention, especially for those experiencing complex trauma. Unfortunately, what constitutes complex trauma is contentious, alongside growing criticisms of diagnostic categories and labels. Recent literature critiques the symptom clusters and diagnostic categories/labels approach compared to focusing on the concrete impacts and functional nature of behavioural responses to trauma in context.

**Aims:**

This research aimed to assess the conceptual maturity of complex trauma for children and young people who have experienced abuse and neglect by discussing the concept with Australian experts. The research aimed to conceptualise complex trauma through a dimensional lens and impacts-based approach. The overall aim was to increase understanding of the development and maintenance of complex trauma and its distinctiveness from other types of trauma.

**Method:**

Group interviews were conducted, and reflexive thematic analysis was used to analyse the data. A member-checking survey helped review and improve the findings.

**Results:**

Findings suggest a vast array of impacts from complex trauma, that diagnostic boxes may not be right for complex trauma, and that the potentially chaotic cycle of complex trauma perpetuates issues. Results from this pilot indicate that complex trauma may be an immature concept for expert clinicians and researchers alike.

**Conclusions:**

Despite assessing complex trauma as an emerging or even immature concept, the discussion generates direction forward and suggests further research avenues. Associated ideas and emerging concepts begin a conceptual discussion of complex trauma.

**Supplementary Information:**

The online version contains supplementary material available at 10.1007/s40653-024-00648-z.

Trauma can result from a range of experiences but most commonly refers to an event that overwhelms the ability to cope and the associated response to the event (Tarren-Sweeney, 2013). More recently, in the Australian Child Protection context (Paton et al., 2023), trauma has been defined as:

*… the psychological*,* physical*,* social*,* emotional*,* cultural and/ or spiritual harm caused by exposure to an event*,* or series of events that are emotionally disturbing or life-threatening. It impacts an individual’s sense of self*,* safety*,* social connection and ways of coping. For this purpose*,* ‘trauma’ can be defined both in terms of an event/s causing harm*,* and the harm that exposure to that event/s causes.*

Exposure to acute and isolated traumatic events, like severe accidents or injuries, is associated with distinct and conditioned responses to related triggers. In contrast, exposure to chronic events, such as child abuse and neglect, is associated with widespread and pervasive effects on functioning (Morelli & Villodas, 2022). Distinct concepts have been proposed for understanding chronic forms of trauma; one such concept, originating from Herman (1992), is ‘complex trauma’.

## Complex Trauma – a Contentious Concept

Complex trauma as a concept is generalised to encapsulate a wide range of phenomena and used interchangeably for the exposure to traumatic events, the experience of multiple or different incidents, and the effects of that exposure or experience (Cook et al., 2005; Wamser-Nanney, 2016). There is agreement that abuse and neglect-related complex trauma in children exist when abuse and neglect are prolonged, repeated, interpersonal/relational, and during early periods of critical development (Cook et al., 2005; Herman, 1992; Morelli & Villodas, 2022; Spinazzola et al., 2018). The literature indicates that the presentation of complex trauma related to child abuse and neglect is a diverse cluster of behaviours or symptoms associated with problems across the lifespan, which, in turn, poses a risk for additional trauma and a cumulative harmful impact on functioning (e.g., impaired occupational, academic, and social functioning; Cook et al., 2005; Ford, 2021).

Symptoms associated with complex trauma can manifest across developmental stages. Symptoms can have wide-ranging adverse effects related to attachment, affect and behavioural regulation, cognitive functioning, dissociation, interpersonal dynamics, and self-concept, as well as impact a person’s worldview and perceptions and beliefs about others (D’Andrea et al., 2012; van der Kolk, 2005). Such trauma has been found to impact transdiagnostic regulatory systems related to social information processing, emotional processing, threat-related processing, and stress and arousal response (Flechsenhar et al., 2022). In addition, there is a breadth of presentations that are found to be commonly associated together, including disrupted eating patterns (e.g., binge eating, hoarding food), sexualised behaviour, and issues related to toileting (e.g., enuresis, encopresis; Tarren-Sweeney, 2008, 2013). Despite this, capturing the full sequalae intended by the term complex trauma has so far been unsuccessful, and the scope and heterogeneity are not necessarily articulated in the current diagnostic systems (Tarren-Sweeney, 2008).

## Diagnostic Challenges

Complex trauma has been implicated in a wide range of psychopathology; however, contention in the diagnostic literature surrounds what constitutes complex trauma without any formal diagnosis with an agreed-upon symptom profile. D’Andrea et al. (2012) argue that a single current psychological diagnosis cannot account for the multifaceted cluster of symptoms associated with complex trauma. For example, the diagnostic criteria for Post-traumatic Stress Disorder (PTSD) in the DSM-5 (American Psychiatric Association, 2013) tends to be too narrow (particularly for children and adolescents) and does not adequately consider the pervasive and developmental effects of chronic child abuse and neglect (Ford, 2021; Morelli & Villodas, 2022). Other common DSM diagnoses administered to children who have experienced abuse and neglect include Depression, Anxiety, Attention-Deficit/Hyperactivity Disorder (ADHD), Oppositional Defiant Disorder (ODD), Conduct Disorder (CD), Disinhibited Social Engagement Disorder, and Reactive Attachment Disorder (Tarren-Sweeney, 2008).

While not formally recognised in the DSM, van der Kolk (2005) proposed a diagnosis of Developmental Trauma Disorder (DTD). The proposed DTD was developed to extend PTSD by encapsulating interpersonal victimisation, widespread dysregulation, and developmental impact, among other symptoms (van der Kolk, 2005). Further extending PTSD is Complex PTSD in the ICD-11. Complex PTSD refers to the experience of PTSD symptomology and affect regulation problems, negative cognitive distortions about self and feelings of shame and guilt, and difficulties maintaining relationships and feeling close (Maercker et al., 2022). These diagnostic criteria have been used interchangeably with what complex trauma ‘is’ (e.g., Lawson, 2017). Although research has provided support for the diagnosis of Complex PTSD in adult populations (Brewin et al., 2017), there is a paucity of research regarding Complex PTSD in children and adolescents (Haselgruber et al., 2019; Maercker et al., 2022).

Although diagnostic classifications provide a standardised language, research suggests they do not capture the full extent and severity of the symptoms and the core factors underlying complex trauma from child abuse and neglect (Ford, 2021; Morelli & Villodas, 2022). Given the significant symptom overlap between the possible range of diagnostic classifications, practitioners have to navigate differential diagnosis considerations and the potential comorbidity of diagnosis commonly associated with children who have experienced abuse and neglect. Providing multiple diagnoses to cover off on the full range of symptoms present, or misdiagnosis is therefore common, with children being labelled as having ADHD, PTSD, CD, and generalised anxiety alongside a laundry list of behavioural labels such as inattentive, disruptive, having poor self-regulation, educationally at risk, developmentally delayed etc. This can lead to issues of pathologising a child’s presentation as comprising multiple significant mental health concerns rather than seeing their symptoms for what they are – a reaction and consequence of the complex interactions of their child abuse and neglect experiences within early childhood relationships.

This misattribution of symptoms to a plethora of other disorders rather than to complex trauma leads to an underestimation of the profound impact of child abuse and neglect experiences. Given that diagnosis and accurate symptom classification and attribution is the foundation of selecting the correct matched intervention, these issues with regard to diagnosis become more fraught. The issue becomes not only the overdiagnosis or misdiagnosis of children in our attempt to ‘categorise’ their presentation but the misalignment with effective treatments to resolve the many symptoms associated with their presentation. For example, if a diagnosis of ODD is made, the treatment of choice may be behavioural management via applied behaviour analysis. However, behavioural confrontation within relationships seen in children with a complex trauma presentation may respond better to a trauma-focused and targeted approach and, in fact, may have their symptoms exacerbated by a purely behavioural treatment response that fails to consider the trauma and relational dynamics at play (Leenarts et al., 2012; Lindauer, 2012; Morelli & Villodas, 2022). Such categorical diagnosis, to the exclusion of other possibilities or consideration of broader symptomatology, can also lead to a situation where only part of the presentation receives a response or treatment. Cuthbert and Insel (2013) posit that categorical diagnostic criteria are associated with reduced symptom specificity and a loss of heterogeneity within symptom presentation. For example, if a child is diagnosed with PTSD or ODD, only these associated symptoms would be considered, regularly assessed and responded to within treatment. Thereby treating only part of the presentation that may be associated with the broader presentation of complex trauma.

## From Diagnosis to Symptoms or Impacts

Recent literature often advocates for alternatives to symptom profiles, clusters, and diagnoses. For example, Guerin (2017, pp. 93–127) critiqued the symptom-cluster or disorders approaches, arguing for a life-strategy approach, where trauma-originating behaviours are seen as functional responses for survival in difficult life situations. Likewise, the Power, Threat Meaning Model of the British Psychological Society situates such behaviour in similar complex contexts and meanings (Johnstone & Boyle, 2018). Given the issues in defining complex trauma and the diagnostic confusion, symptoms- or impacts-based approaches independent of a stand-alone definition or diagnosis may be warranted. Conceptualising complex trauma based on symptoms/impacts provides a framework that encompasses the multifaceted reality and is sensitive to the aetiology of complex trauma.

Further, adopting a symptoms- or impacts-based approach provides an opportunity to build upon existing therapeutic approaches. Symptom approaches are in accordance with the International Society for Traumatic Stress Studies (ISTSS), which suggests interventions be client-focused, flexible, components and phased-based, and targeted specifically to symptoms (Cloitre et al., 2012). In addition, Ford (2021) posits that increased specificity in characterising target symptoms and the treatment mechanisms may be required to support post-traumatic growth and facilitate adaptive functioning.

The broad aims of this exploratory pilot study are, therefore, to (1) adopt a dimensional and impacts/symptoms-based approach to the conceptualisation and formulation of children with abuse and neglect-related complex trauma and (2) increase understanding of the development and maintenance of child abuse and neglect related complex trauma and what makes it unique from other forms of trauma. Informed by the broad aims of this study, this research was guided by the following research questions:

From an expert perspective:

1. What impacts and/or constructs do children and young people with complex trauma from abuse and neglect experience/present with?
2. How do these impacts compare with current diagnostic criteria, and are they reflected holistically in any one diagnostic tool?
3. Can these impacts be grouped into symptom clusters/categories?
4. What makes complex trauma from child abuse and neglect distinct from other types of trauma?
5. What factors influence the development of complex trauma in children and young people who have experienced child abuse and neglect?
6. What mechanisms serve to maintain complex trauma presentations into adulthood for some children and young people who have experienced child abuse and neglect?

## Methods

### Study Design

This research used a qualitative, constructivist/interpretivist exploratory, expert group interview design (Krueger & Casey, 2015). The approach allows for an array of information to be collected with the agreement or disagreement between participants to provide confirmability and trustworthiness to the data. After completing the group interviews and analysing the data from the group discussions, participants were invited to complete an anonymous online survey to member check the data and analysis. The study is best considered a pilot investigation and exploration of concepts, given the limited sample.

### Procedure

This research was approved by the [redacted]. Purposive and snowball sampling techniques were used, with potential participants recruited from the research team’s professional national networks and contacts. Potential participants were sent an email invitation, including an information sheet about the research project. Participants who expressed interest in the study were then provided with a link (hosted by Qualtrics) to read the information sheet, complete an online consent form, and provide demographic information. If participants had not completed the online consent before the group interview, verbal consent was also obtained, and participants were asked to complete the online consent and demographic questions after the session. The group interviews were conducted over four weeks in November and December 2022. The group interviews were conducted via videoconference using Microsoft Teams recorded using the audio-visual recording and live transcription functions. The group interviews ran for one-and-a-half to two hours, depending on the number of participants present and the collaborative nature of the discussion.

The structure of the group interview was in line with a focus group schedule designed to aid consistency across the groups. However, the facilitators largely facilitated the discussion rather than the conversation being between participants, hence the change of term to group interviews. Each group interview discussion included the provision of informed consent, welcome and introductions, a brief background summary of the research project and data collection, discussion regarding the research questions, and conclusions. The group interviews consisted of one primary facilitator ([redacted]; consistent across all groups), a co-facilitator ([redacted]), and a minimum of two and a maximum of four participants across groups to allow all participants to speak in the time. Following the group interview discussions and data analysis, a summary of the themes was generated and incorporated into an anonymous online survey. Participants were sent an email with a link (hosted by Qualtrics) that directed them to complete the survey to member check the results (Birt et al., 2016) and provide comments for improvement.

### Participants

The inclusion criteria were that participants be above 18 years of age, reside in Australia, and have practice experience in child abuse and neglect and/or complex trauma from child abuse and neglect. Given the pilot nature of the study, a small recruitment pool of 27 individuals was invited to participate in this study; 17 expressed an interest (a response rate of 63%), with three unable to attend the scheduled group interview session. Four participants scheduled to attend the group interview discussions could not attend on the day, resulting in ten participants.

The ten participants were professionals with experience working within child protection systems, clinical practice, specialising in the child trauma sector, and/or demonstrated knowledge of children and young people who have experienced trauma from abuse and neglect (clinical psychologist *n* = 5, forensic psychologist *n* = 1, psychiatrist *n* = 1, academic *n* = 1, occupational therapist *n* = 1, and therapist/ manager for a therapeutic service *n* = 1). Five participants were currently working in non-government organisations, two in government organisations, two were self-employed, and one was working across both government and non-government organisations. The overall length of professional experience (all roles) for participants ranged from three years to over 21 years, with the majority having 13 or more years of experience overall (60%). Participants were aged between 25 and 29 and 69 − 64 years (ranges were asked for). Most of the sample identified as a woman or female (90%; 10% man or male), and most identified as having a non-Aboriginal Australian cultural background (80%; 10% British; 10% Netherlands). All participants resided in Australia, with three each in the states of Western Australia and South Australia (30% each), two in Queensland (20%), and one each in the Australian Capital Territory and Victoria (10% each).

### Data Analysis

The main analyst ([redacted]) was provided with the group interview transcripts and removed identifiable details. Names and identifiers were replaced with markers. In this process, the Qualtrics survey attributes (e.g., profession and length of time in that profession) were anonymised and tied to these markers in NVivo as cases. All transcripts were imported into NVivo, and a mixture of deductive codes – based on team discussions and previous knowledge of complex trauma – and inductive codes – open/eclectic coding processes deriving meaning units from the transcripts (Saldaña, 2021) – were used to organise the data. A constructivist/interpretivist approach to Reflexive Thematic Analysis was the primary analysis for this data (Braun & Clarke, 2021). However, the analyst ([redacted]) acknowledges the influence of critical approaches from Social Contextual Analysis (Guerin, 2016) and psychological Discourse Analysis (Potter & Wetherell, 1987) on the coding and theme generation with comments where appropriate in the results and discussion. The results are themes generated by the analyst.

A second coding pass was completed to confirm that the codes and themes were uniform throughout the data. The three themes were generated using the research questions as a foundation, with the codes and concepts found in the group discussions. As per Braun and Clarke’s (2021) guidance for Reflexive Thematic Analysis, there was consistent reflexive noting and discussion between the analyst and the research team as to the reason, feelings, and reactions to the codes and to seek understanding between the different reflexive positions of the research team. It is worth noting that the main analyst is not a clinician with direct experience in treating complex trauma; this is seen as a strength for analysis as they approached the coding in an open/naïve way while the rest of the team have clinical experience with the topic.

Finally, the member checking data (constructivist approach on synthesised analysis, see Birt et al., 2016) were incorporated directly rather than a third formal coding or analysis phase as the agreement on the themes was high, and the suggestions for change were direct and minor. Such changes are noted below where needed. A report by [redacted] was generated on the first pass coding, which can be reviewed for the changes made through member checking. The quotes below are presented in an ‘intelligible’ verbatim style. Some repeated words missed words, or pauses have been added, removed, or included to allow the quotes to flow intelligibly and allow for understanding without utterances before, after, or interjections from other speakers.

## Results

Three broad themes were generated using the Reflexive Thematic Analysis (Braun & Clarke, 2021). These themes are broadly conceptual answers to the research questions. The three themes weave in-between one another and are not exclusive in process or kind. The three themes were rated as generally extremely accurate of the view of the participants in the member checking survey. Some discussion points, particularly of a discursive analytical nature, are present throughout the results.

### Theme 1: Any Impacts or Behaviours are Valid, and any Impacts are Possible (RQ1)

Initially, “the many symptoms of complex trauma” was reported here, but comments in the member checking survey changed this language to impacts. Many impacts or behaviours and clinically significant constructs were provided for presenting complex trauma. While many impacts were provided, their inclusion was accompanied by comments explaining the difficulty of generalising or narrowing complex trauma down to a simplified presentation of impacts or constructs.

#### Participant 2.1

*Complex trauma is like shifting sand like it can, it just, it can be really dynamic and look really different. Yeah, and you wouldn’t want kids missed, I guess.*

#### Participant 3.2

*What we have in place at the moment is really insufficient and that makes what is already really complex work, I think, even harder when there isn’t a clear… [It is] very confusing if we’re confused.*

**Fig. 1:**
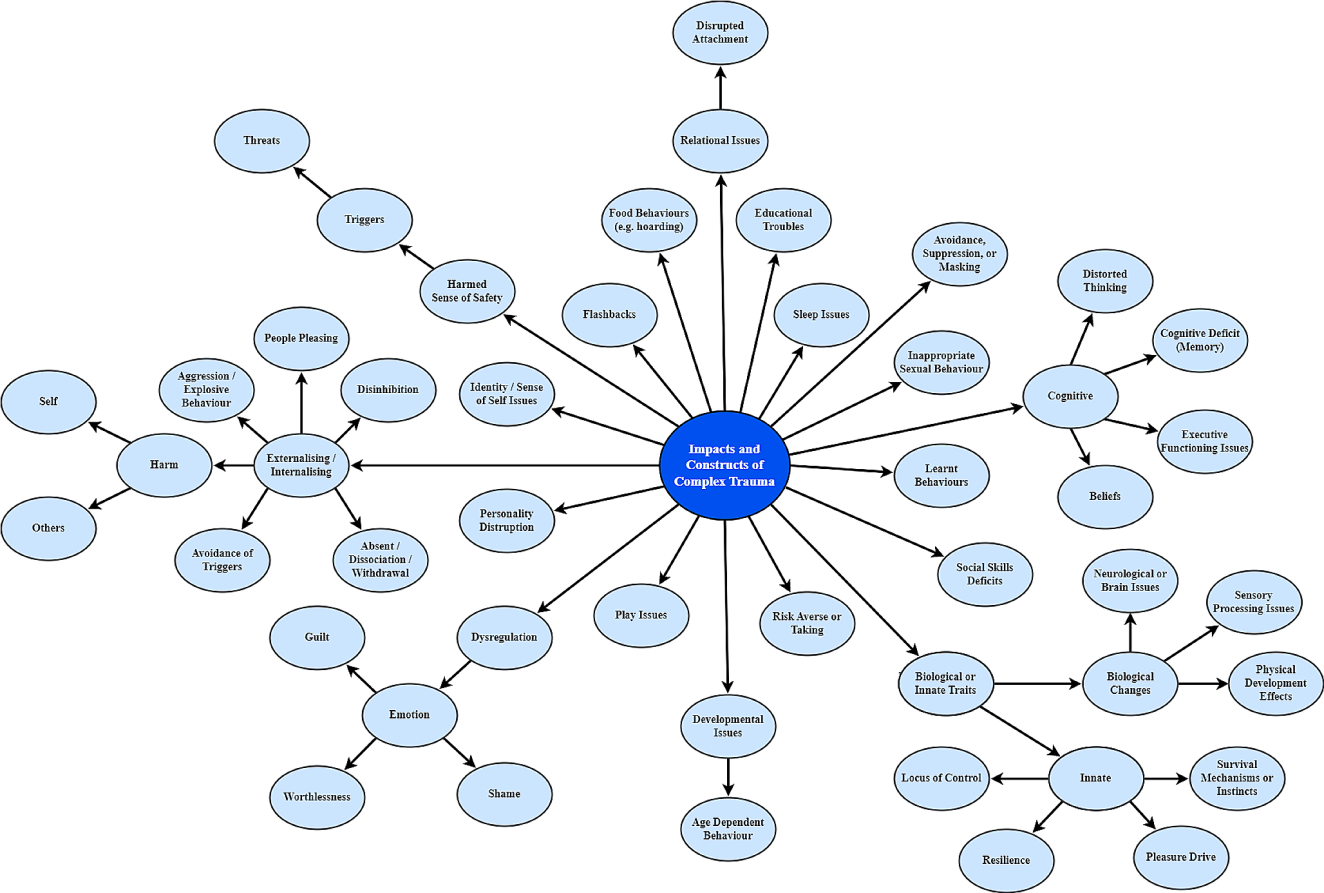
An array of the impacts and constructs related to complex trauma, as described by the participants

Despite the difficulty in narrowing down the vast array of impacts and constructs, relatively similar discussions in the four group interviews indicated that this array is agreed upon. It may seem that this answer is not overly helpful for the research question, given the array of impacts is so varied; yet this reinforces the many symptoms present in the literature (e.g., D’Andrea et al., 2012; Tarren-Sweeney, 2013; van der Kolk, 2005). While 49 different impacts, their variation, and constructs were discussed, only 19 were accompanied by examples or descriptions, while others were only named. Figure 1 is the array of impacts of complex trauma confirmed and adjusted through member checking (9/10 agreed, 1/10 neither agreed nor disagreed with the array).

Overall, there are many different presentations of complex trauma. However, within that variation, there was a message that focusing solely on the presentation at a particular time was not always correct. Rather, varying and changing impacts are common and should be expected, given the cycles that complex trauma and the systems around it create. Often, a significant variation of impacts appears over time, influencing a range of functional and developmental domains. For example, presentations in early childhood may be marked by self-regulation difficulties, inattention in the classroom, difficulty engaging with peers, and aggressive behaviours towards others, while later in development, presentations may be marked by sleep disturbance, sexualised behaviour, and various self-harming behaviours.

### Theme 2: No One Box for Complex Trauma (RQ2, 3)

Many group interviews discussed current, former, and future diagnostic criteria for understanding and treating complex trauma. Throughout the discussions, one consistent notion was repeated: that complex trauma, to date, fits no one box for diagnosis and that no criteria these professionals have seen thus far include the right content to capture the complexity described.

#### Participant 4.1

*… If you look at the presentations and adults that need help and get a diagnosis, the majority of them have trauma and we have nothing really; like the DSM is symptom based. It’s a checklist of symptoms, so that doesn’t really fit with this. I find it even unhelpful ‘cause there’s now like a tendency to, you know, there’s more knowledge about PTSD, but then when you don’t meet the criteria, you don’t get treatment.*

Unfortunately, the benefits of diagnosis were also reported to be imposed rather than particularly about the presentation or the issues that clients experience. For these participants, as professionals, the diagnosis was more about ensuring that clients could gain compensation, funding, or access to treatment, even if the diagnoses were not entirely accurate.

#### Participant 3.1

*But there is such variability within that that I think then it opens the door for or let’s call it ASD [Autism Spectrum Disorder] because then we’ll get the NDIS [National Disability Insurance Scheme] package that we won’t get if we mention the word trauma which is a terrible position to be in, I think, because you know all we’ve talked about today is there is something different about trauma within care giving.*

The presentation of entrenched complex trauma issues was consistently commented on as changing throughout the lifespan and depending on external and contextual factors.

#### Participant 4.2

*Because the trauma, the complex trauma is going to emerge as personality disorder, and you don’t actually see it until their teen years. So, you get these kids who are, there’s a, if you like, the traumas derailed their personality development.*

The issue of diagnosis and changes over the lifespan was pointedly described, given that the nature of diagnosis shifts drastically as a child matures. The participants highlighted that those common diagnoses were ASD and ADHD, but many more were present. In adolescence, these may start to present as emergent personality issues through the development of substance use issues and personality disorders (e.g., borderline personality disorder).

#### Participant 4.1

*They can have any diagnosis that you can think of because it can look like ADHD. It can be both. It can look like autism; it can be both. It can look like an eating disorder, and they can use substances.*

At the same time, sometimes the diagnostic criteria were too narrow, and the child may not fit into any box while displaying many different issues.

#### Participant 2.1

*You know, running with this idea of having clusters and stuff that you know, I know with, you look at other, other disorders, like you know, Autism or ADHD and things like that. And you’ll get, you get children and young people who clearly are presenting with things that are representative of that issue, but because they don’t quite meet some kind of part of a cluster or category; it’s wiped and it’s not a thing for them. And so, I think there is a danger in, like, if it was, if we were using things like this for diagnosis, that if a child didn’t meet one particular bit, or you had whoever was doing the assessment was not skilled enough to recognise bits of what that child was presenting with as representing a certain part, that child would fall through the cracks.*

**Fig. 2:**
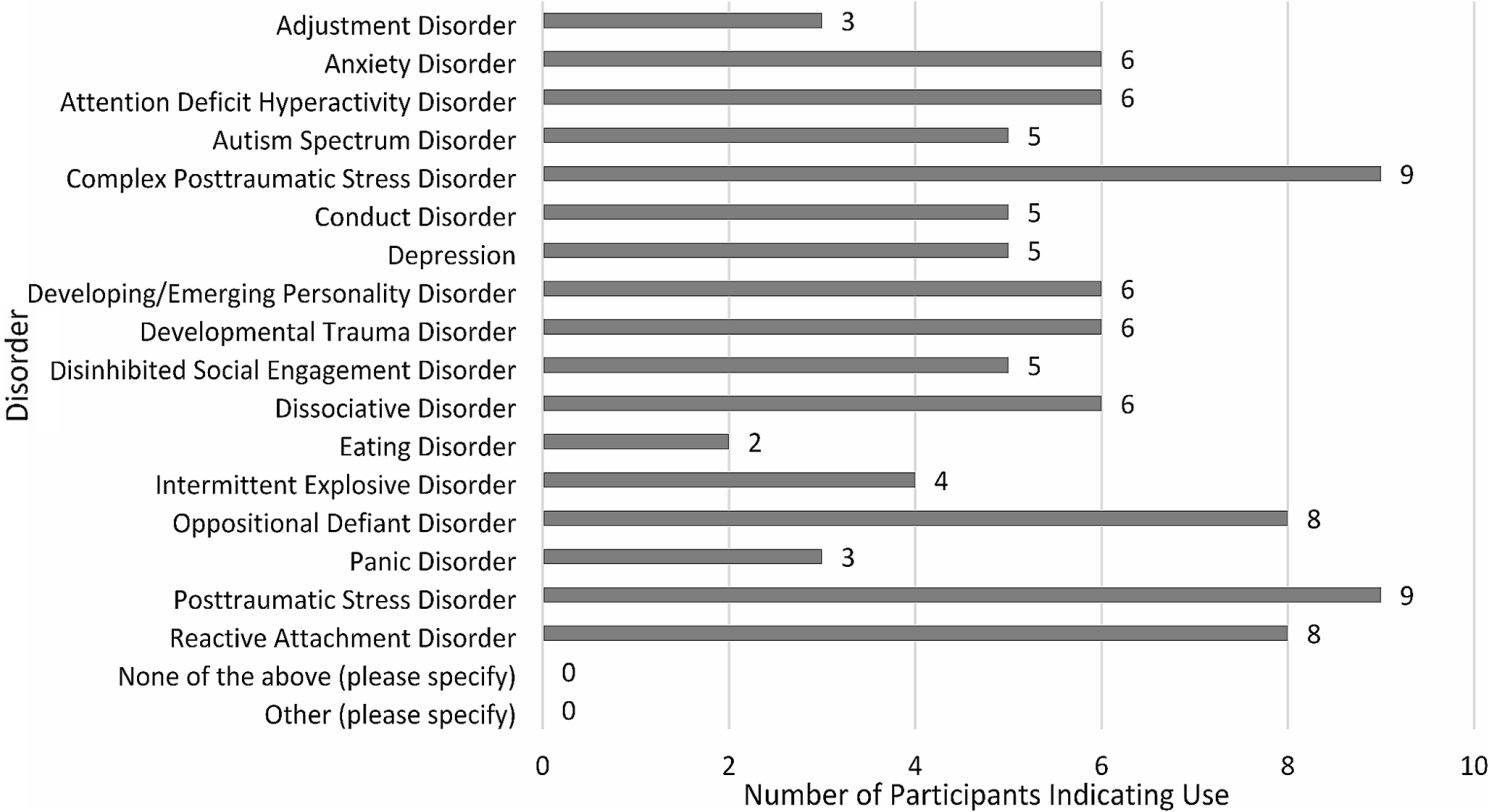
Number of participants indicating the use of a disorder for the presentation of complex trauma. *Note*. Borderline Personality Disorder (BPD) was mentioned in the groups but was missed in the list. We assumed participants would add BPD under the “other” function but did not. Potentially BPD and other disorders are used, but time to add disorders in the survey was an issue. Otherwise, potentially, “emerging personality disorders” may be more appropriate for children

Within the member checking survey, participants were asked to indicate the disorders they had used or seen used concerning complex trauma; the list was populated by disorders mentioned in the interviews. Figure 2 below shows the disorders used.

Consistently, participants reported that the reality of complex trauma does not fit the functional imposition of welfare, protection, and compensatory systems. Presentations may not be direct and are rather reported by teachers, parents, or other caregivers; if a behaviour is not dysfunctional or disruptive for adults, a child could *“fall between the cracks”*. Such missed cases were those that internalise and suppress symptoms while still having attachment or other regulative emotional issues. These individuals may even present with worsened symptomology through therapy as the suppression of these problems and related behaviours is overcome. It is possible that as a child engages in therapy and learns ways to connect with and express their emotions and thoughts within a safe, open, and non-judgemental relationship, they connect with their hidden distress and share it.

#### Participant 2.1

*The kids who internalised are often they’re the ones that you miss quite easily. Teachers won’t pick up on it because they’re really quiet and they don’t cause trouble. You know, parents often don’t pick up on it because you know they’re so busy with their own lives that you know, if the child isn’t actively, you know, bringing the house down with drama.*

#### Participant 4.1

*A lot of the young people that I work with also feel that in as “No, no, no, no, no, I feel fine, not a problem”, so then when you, after treatment they score higher [on symptom ratings]… they also become more aware of their feelings so they can be like feeling. I say feeling more, so, also scoring higher.*

The participants did find that understanding trauma through constructs and symptoms – including diagnoses – could be helpful for treatment.

#### Participant 2.1

*And the diagnosis isn’t my focus. And my focus is reducing that symptoms to optimize their functioning.*

#### Participant 4.1

*Yeah, I would. I would say yes, we need those criteria because to be able to say, okay, this person needs that kind of treatment, and that person needs that kind of treatment.*

Unfortunately, systemic problems compound issues around treatment and diagnosis or the classification of trauma. Foremost were consistent comments about the lack of real support given by the systems in place for children’s protection. For example, the transient nature of workers in out-of-home care can compound common issues from abuse and neglect like disrupted attachment.

#### Participant 3.1

*Unfortunately, you know, we see that in children in care. They do get really abused in. Yeah. Yeah. In a range of settings.*

When labels are used, participants could render appropriate treatments and supports, but pervasive issues of stigma and misunderstanding are often created by these labels:

#### Participant 1.2

*You know things that teachers are scared before they even meet the kid because they’ve got this rap sheet of 17 different diagnoses of intermittent explosive disorder, ADHD. Yeah. And I think that that’s really problematic, but my flip argument to that is that. That’s how they get funding.*

#### Participant 4.1

*I think for these kids we need diagnostic criteria to provide them with the treatment they need, and I would rather look at it that way than saying this is your label and that’s your identity now because a lot of kids that are diagnosed with something that it then becomes their identity like borderline personality or whatever.*

The issue of stigma can occur without formal labels as the behavioural presentation of the impacts of complex trauma often are primarily the negative reactions of other people.

#### Participant 1.2

*Yeah, it’s now that because he’s this trauma kid and his behaviour that he’s displaying that is even remotely kind of. I hate it, it’s not even sexual, like, like anything that’s remotely even the slightest bit, because he’s this trauma kid. It’s now a harmful sexual behaviour. So, it’s like where we take that too far and start pigeonholing these kids based on this understanding of their trauma history.*

#### Participant 3.3

*The problem for children who’ve experienced trauma is all of the adults around them trying to make sense of what essentially is a cluster of behavioural presentations that are challenging for adults.*

The participants often responded to these diagnosis issues with alternatives to the current system. What was mentioned reverberates with current international trends that are critical of diagnostics (Guerin, 2021; Johnstone & Boyle, 2018). Some of the participants’ alternatives included transdiagnostic approaches, including a dimensional model distinguishing threat-based experiences (e.g., physical abuse) from deprivation-based experiences (e.g., neglect; McLaughlin et al., 2014).

#### Participant 3.4

*And I don’t know whether anybody has really tried to sit down and match those to a case of a child that has experienced, and I think we, you know, I personally am very persuaded by the dimensional model that we have to differentiate threat from deprivation. And so, if we if we take that as a starting point sequela of extreme deprivation versus extreme threat, and that then I’m thinking like, well, what is complex trauma anyway? So, I’m really just all I’m throwing in is a is a whole series of kind of messy thinking because that’s sort of where my thinking is at the moment. It’s confuse’ by trying to integrate.*

The participants were also critical of the internal or dispositional attribution that disorders and diagnoses often create. These children were exposed to chronic abuse and neglect and reacted to it. Resonating with the systemic and often oppressive nature of diagnosis, participants were keen to highlight that the children and their behaviour were not the crux of the problem, but rather, they were responding to a problematic care situation with the only afforded behaviour they had at their disposal:

#### Participant 3.3

*I like the idea of a complex trauma diagnosis because it locates the problem outside the child as something that’s been done to them. And this is kind of the consequence of what’s occurred….*

These findings reinforce criticism of current diagnostic-based practices, taking that criticism further than expected. The outcome of such pervasive critique is that we may need to rethink current approaches to complex trauma presentations and treatment.

#### Participant 3.1

*I would, I would agree. I think we’re at, our current system is incredibly inept, inadequate.*

**Fig. 3:**
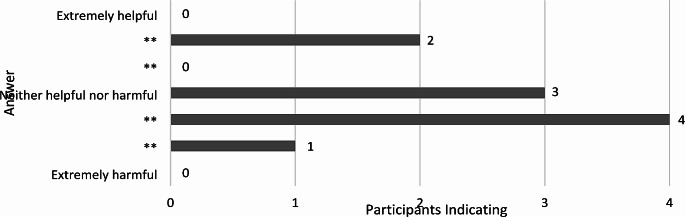
Participants indicating diagnoses help or harm to children’s wellbeing

The member checking survey asked the participants if a diagnostic criterion or a conceptual framework was needed for complex trauma – all participants affirmed that it was needed. Participants were then asked if diagnoses were helpful or harmful to children’s quality of life. Figure 3 shows the spread of these responses, which are best understood through the comments accompanying:

Participants gave several reasons for their scores, including:

- *Children can overcome trauma; this label can give the impression this is stuck for life.*
- *Rarely helpful but can occasionally lead to an appropriate intervention. Harmful as some diagnoses are lifelong.*
- *To help communicate with courts and agencies.*
- *It is individual. A diagnosis can increase access to funding and support. However, a diagnosis can also lead to the underlying issue (trauma) that is causing the symptoms that led to the diagnosis, being ignored.*
- *The systems we work in rely heavily on diagnoses for support to be given.*

In particular, to this point, we must acknowledge the diverse way diagnostic issues compound, especially for minority groups for whom the association with diagnosis can become essentialistic myths. As authors, we would note the example, in the Australian context, that the lives of Aboriginal and Torres Strait Islander peoples may be misunderstood by diagnostic abstraction. Critiques from Indigenous and Feminist scholarship highlight that the diagnosis approach aims to bring problematised behaviours together as ratified clusters and is made so with white-western and patriarchal lenses, which do not account for the on-the-ground power, threats, and meaning-making of the people often at the mercy of these systems, especially at the point of multiple intersections between race, economic status, and historical injustice (Dudgeon & Walker, 2015; Gee et al., 2014; Guerin, 2021; Johnstone & Boyle, 2018). Exploration into complex trauma for Aboriginal and Torres Strait Islander people with the Noongar people of Western Australia highlighted the root cause as the pervasive effects of colonisation producing loss and grief, fractured connections, and layers of abuse. Continued harm to Aboriginal peoples through the stolen generation, family disruption and social effects of poor mental health add to the layers comprising complex trauma for First Nations people (Hovane et al., 2023). We must be sure that progress forward does not become further tools of oppression, as reflected in recent efforts to emphasise culture, racism, and discrimination in the DSM-5-TR largely by shifts in language but also variation of symptoms based on “cultural norms” (American Psychiatric Association, 2022). Most important to understanding the application of diagnosis across heterogeneous groups is that they provide a uniform and equitable function in context.

Given the above points, participants were then asked to endorse or add possible benefits to the use of diagnosis in the case of complex trauma; these elements, in frequency of endorsement order, are included in Table 1:

**Table 1:** Endorsement of positive reasons for diagnosis (in frequency order)

Endorsed Element	No.
Provides support for intervention choices	8
Provides structure for understanding	8
Allows for funding opportunities (e.g., NDIS)	7
Allows for policy decision-making	7
Provides support for research (funding)	7
Provides support for research (structure)	7
Provides structure for training	5
Validates the experience for those experiencing it	5
Provides uniformity when complex trauma is discussed	4
Other *“Communicating with courts”* *“Builds empathy and understanding of stakeholders*, e.g.,* teachers”*	2

There was also a final comment by a participant to this point (member checking survey):

#### Participant

*Although there are many pitfalls to the diagnostic system in general - while complex trauma is not appropriately captured in a “diagnosis”*,* children and young people continue to be failed by systems and not provided the supports they need/deserve because unfortunately systems and funding are driven by diagnoses.*

### Theme 3: The Chaos of Complex Trauma (RQ4, 5, 6)

When differentiating between trauma and complex trauma, a few interesting though complicating considerations were raised in the group interviews. These included comparisons between other types of trauma, whether to focus on impact or experience, changes in experience through the lifespan, and the cycle of complex trauma.

#### Comparison between Types of Trauma (RQ 4)

Within the discussions, other types of trauma were contrasted or compared by the participants. This included differentiating between the common instigation of complex trauma – often chronic abuse or neglect – and the typically episodic trauma from natural disasters or medical trauma. However, participants admitted these could also be complex if multiple. The separation was not specific or exact; it moved back and forth between multiple-trauma and time-based conceptualisation without a reliable operationalisation.

A more complicated set of inclusions were developmental and relational trauma, which concepts both directly related to the cycle of complex trauma as described amongst all group interviews (see below). Importantly, there were comments that due to a lack of definition for complex trauma, developmental and relational trauma overlapped conceptually, some stating they used the terms interchangeably:

##### Participant 1.1

*I thought that [complex trauma and developmental trauma] were the same, but genuinely thought the concepts were interchangeable….*

##### Participant 1.2

*I feel like developmental trauma is always complex, but complex trauma isn’t always developmental.*

This idea was present throughout group interviews:

##### Participant 3.1

*You know, we all use different, you know, complex trauma, developmental trauma. I think we’re all. You know, meaning the same thing. But there is such variability within that.*

Within the member checking survey, participants were asked to state if developmental trauma, relational trauma, and complex trauma were distinct concepts/presentations. The results were rather inconsistent, with some indicating that these constructs were not distinct while others indicated they were highly distinct. This reinforces a point; professionals may or may not intend the same thing when using any of these terms. The lack of intended uniformity was suggested in the group discussions for the term complex trauma; potentially different concepts are ultimately at play, despite expertise.

##### Participant 4.2

*If I said complex trauma in a report, and Bessel van der Kolk said complex trauma and the local psychiatrist say complex trauma; I’m not sure we’re even all talking about the same thing. And we’re probably not.*

Furthermore, linking the experience of abuse or neglect with the resultant behaviours and, therefore, the disorders presented with issues. The previous confusion between event and effect, or experience and result, was once commented on rather starkly as just ‘made up’:

##### Participant 4.1

*Complex trauma refers to what you have experienced, whereas complex PTSD refers to the result of that. And developmental trauma disorder is the result of complex trauma and then there’s chronic trauma and relational trauma. So relational, personally is complex. No one knows. It’s just made-up, yeah.*

Within this there were attempts to separate the idea of complex trauma from single instances of trauma, for example, in medical or natural disaster events:

##### Participant 3.2

*Umm, you know if someone else. If someone experiences another type of trauma, like this is probably not a great example, but like a natural disaster or something like there’s an endpoint to that or there’s like a point where things may begin to return, back to what feels like a normal level, but for children who experience complex trauma this is ongoing, no endpoint.*

##### Participant 4.2

*Yeah, I mean, I’m not sure what I mean can you have complex trauma without the attachment stuff? Umm, maybe it’s not complex, but if you’re left with simple is it, what does that mean? It seems it is simple trauma, if it’s not complex, but having 20 medical operations and whatever, that’s not simple either.*

Complex trauma could, however, be separated from these other types of trauma by the presentation of multiple instances or cases of abuse or pervasive neglect.

##### Participant 4.2

*Well, I think they get used by different people to mean similar things. And I think at some point we really need to identify are they the same or are they different? And I mean complex trauma. At a sort of vague, general level means that someone had multiple traumas over time, sort of thing as opposed to developmental trauma where it it’s really implied that it had traumatic things, but it’s particularly family-based things.*

Ultimately, it seems that complex trauma is not a unique and separate concept, construct, or tool for assessment or analysis for these professionals. Rather, other elements of trauma, context and well-being are considered as they would for other types of trauma. Those other elements are in a unique constellation or cyclical generation that maintains trauma. Experiences of multiple types of abuse and neglect throughout early development were suggested to continue with compounding effects throughout childhood. While such experiences may be explained the same way by experts, they may be labelled with different names, such as developmental trauma, complex trauma, and relational trauma.

#### The Development of Complex Trauma (RQ 5)

Several elements arose in the discussion about what may be key to developing complex trauma compared to other types of trauma. As mentioned above, the instigating factor played a large role in determining whether someone developed complex trauma. Similarly, other factors, including age, meaning-making, the multiplicity of trauma, and the pervasive severity or multidimensional effects, were key to understanding the development of complex trauma, for example, being the (perceived or real) target of abuse.

##### Participant 2.1

*Often it’s because they’ve been targeted within the abuse, or they’ve borne the brunt of it. You know, either by their perception or in reality.*

Otherwise, it seemed important that trauma was occurring at a key developmental stage which lends itself to being pervasive – as a defining feature of complex trauma.

##### Participant 1.2

*I agree with Participant 1.1 in that I still see it as developmental in some capacity, but I do think there’s a distinction between, because it’s developmental in the way that you know and when we’re teenagers we learn so much about the world and our place in it. And if we’re experiencing a period of trauma there, I think she’s right in that you know that that looks very different to experiencing that period of trauma at 30 or 40. But I do think there’s a distinction between that kind of foundational met needs stuff and it occurring when you’re 16.*

Meaning-making was also a consideration for the development of complex trauma and could explain why some children and young people experience the same events but do not experience the same outcome in the presentation of complex trauma.

##### Participant 3.3

*[explaining that a client didn’t know what happened to her was wrong until she saw counter reference points with friends]…then the symptomology came out because she had made sense of her experiences in a different way than she had made sense of them before. So, I wonder how much that meaning making process contributes to the development of what we would say, see, as a cluster of symptoms for complex trauma.*

Instability and unpredictability of the relationships involved in and around the trauma were highlighted as a common context for compounding complex trauma.

##### Participant 2.1

*Thinking like somebody who might be exposed to some really terrible instances of abuse, but there is stability in their life, at least it’s predictable. They can come up with ways of coping with that crap existence versus someone who’s got really crappy experiences happening to them, but everything’s out of control and there’s no predictability and there’s no routine, even if it’s crappy routine, [someone without predictability] is probably likely to be worse off in terms of how they end up.*

The importance of having positive relationships to buffer the trauma and short-circuit issues before they develop into complex trauma was also highlighted.

##### Participant 1.2

*And within that, I think the presence of other safe adults is really, really important. So, you know, Mum may be crap. But Auntie is fantastic, and I think we underestimate the importance of those other kind of safe people around our kids to kind of modulate the impacts of that trauma.*

These varied factors were the aspects that participants in the group interviews highlighted most as those that define the complex trauma experience, despite the varied presentation. Ultimately there was a balance between the idea that complex trauma is defined by its initial instigation factors – be it the abuse type, amount, or timing – and the follow-on impacts of these experiences – changing the meaning of experiences or sources of support. This leaves this analysis to a point where complex trauma is both the experience of traumatic events, which are complex but also is the complexity in the pervasiveness and multidimensional effects of that trauma, even if the instigating event alone does not seem overly complex.

#### Changes through the Lifespan (RQ 6)

Participants were also keen to point out that there is no expectation that the presentation of complex trauma is the same throughout the lifespan. Some pointed out the ability, affordance, and opportunity for different aged individuals to behave in certain ways and how coping strategies develop and adapt over time:

##### Participant 3.3

*Across the, you know kind of developmental stages in that way. I kind of think the behaviours that we see would be developmentally appropriate for someone in distress at that particular developmental stage. So, an infant, can’t you know, run away from home like an adolescent. All they can do is become distressed or shut down.*

In other cases, the behaviour is more likely to change because the triggers for negative reactions change throughout the lifespan, indicating the entanglement of context and experience.

##### Participant 3.2

*I’m certainly going ebbs and flows; I think certainly I’ve seen around different like key developmental points. You know, I think often you know, it’s particularly for children who might have experienced sexual abuse. You know, the idea of engaging in intimate relationships, all these other experiences that they might have throughout life.*

Some core aspects are so persistent and pervasive that they can follow people throughout all the stages of their life, ultimately hampering development throughout their lifespan:

##### Participant 4.2

*Yeah, but it’s also lifespan. I mean, having been a psychologist in these systems now for 35ish years. I saw kids in juvenile justice when they were naughty boys. They then graduate, I saw them in the adult system when they were dangerous men. Then I’ve seen them in family court or, as parents, protection when they were hopeless parents, when I’ve seen him in family court, where they’re now in their 40s and then they’re not so bad that they’re care and protection anymore, but they’re, there’s still, you know, generating because of their personality dysfunction, warfare, and this trail of trauma through their life.*

One presentation of this throughout the lifespan was the lack of trust in others due to those issues with safety and relationships early on in life, compounding into self-fulfilling tendencies to avoid relationships with others.

##### Participant 2.2

*[They] don’t feel like others can be trusted and relied upon, and then if they’ve had multiple experiences of that, whether it’s in the initial abuse experience or just further vulnerability and in their friendship for their first relationship, they potentially are vulnerable to further harm. And then they get this kind of, piling on effect almost all of their experiences and in relationships and feeling that others can’t be trusted. And the way often, if they have learned to cope in a maladaptive way that might push others away or not lead to potentially healthy relationships. Then it just creates further experiences that concern those beliefs that others can’t be trusted.*

### The Cycle of Complex Trauma (RQ 5, 6)

As seen above, a consistent notion arose throughout the group interviews – the cycle of complex trauma. A key aspect of complex trauma is that it is not singular but pervasive. The cycle starts with early but often multiple or pervasive trauma experiences; these are often punctuated by the absence, neglect, or inability of key relational figures (i.e., parents or other caregivers) to be present as stable attachment figures for appropriate regulation to occur (other-, co- and self-regulation). Without these skills and this knowledge, or the continuing context of these issues, both abuse and neglect, there is little for children to understand as counter reference points, and few (if any) positive examples in their life to understand what should be expected or what they can ask for to assist them in their emotional life and to meet their needs.

#### Participant 3.3

*And that there’s not kind of a stable base to start from saying the world is a safe place. What children have learned from a very young age, sometimes from the very first experiences, is the world is not a safe place. And I don’t have anyone to talk to about that or to help me feel safe. For me, I think that’s what’s at the kind of centre of the difference between trauma and complex trauma.*

Systemic issues were also highlighted as central to complex trauma:

#### Participant 1.2

*Path is the wrong word, but you know what I’m trying to say. Like there, there’s those, you know, personal factors, but I think there’s huge, huge systemic issues with reinforcing trauma and adding cumulative harm to these kids, who have already experienced so much.*

#### Participant 2.2

*I feel like when those symptoms that they start to develop don’t fit nicely within society and what is expected of them, it’s even more likely to get that cycle effect. So, we just talked about because, umm, it further breaks relationships and can cause further harm.*

Participants acknowledged the unfortunate realities that the systems of protection are often also sources of continuing issues for these children and young people due to further attachment and regulation issues from rotational and transient relationships:

#### Participant 3.1

*And then if they’re not in foster care and they’re in residential care, they’re really in strife because they’re having to relate to, you know, rotational carers.*

#### Participant 3.3

*That’s right, who use language that demonstrate to them or reinforce to them over and over that they’re not there to be in relationship. They’re here on a shift. They’re working, you know, all those sorts of things.*

**Fig. 4:**
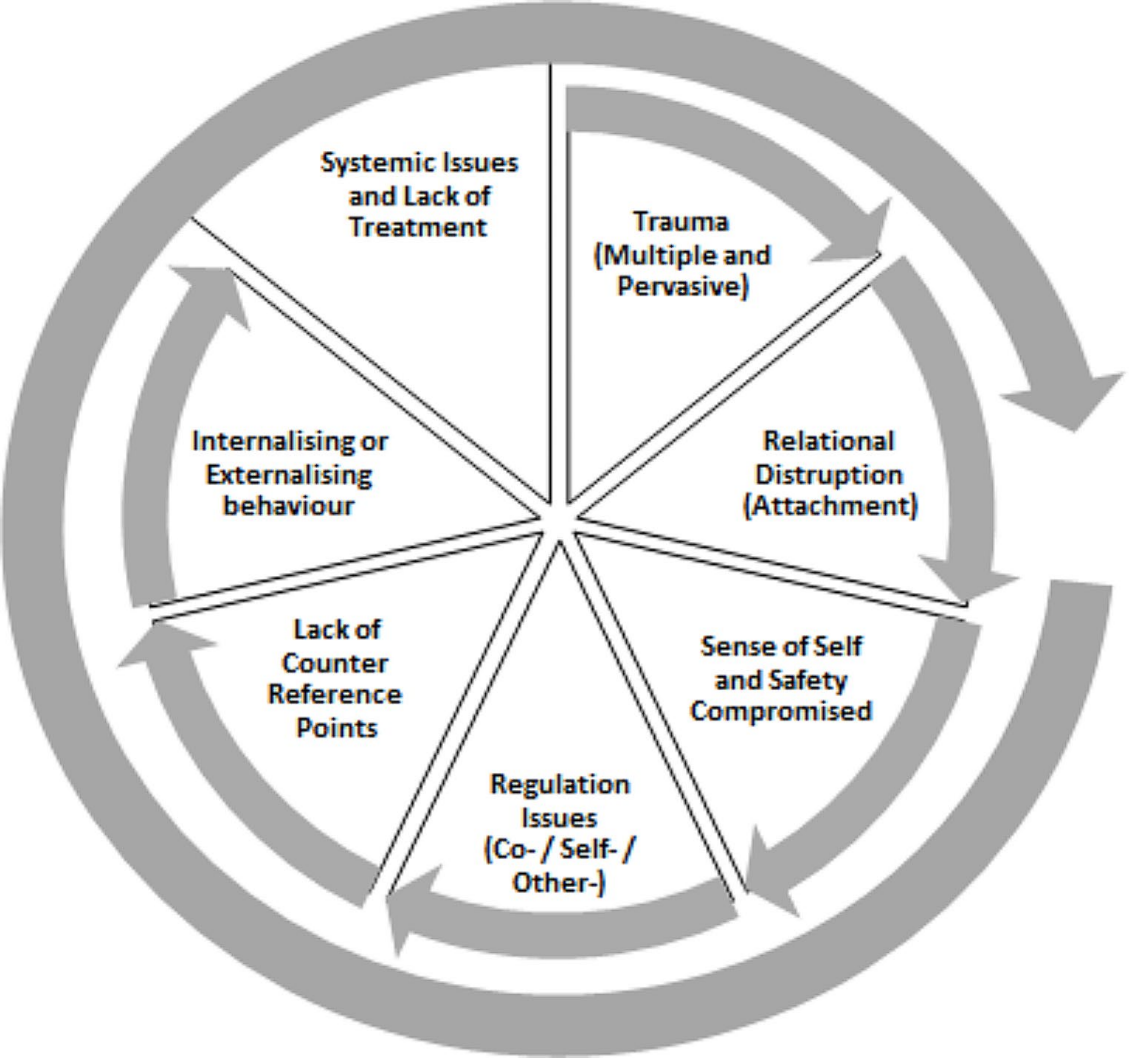
The cycle of complex trauma impacts and constructs

Figure 4 below is a representation of the “cycle of complex trauma”.

**Fig. 5:**
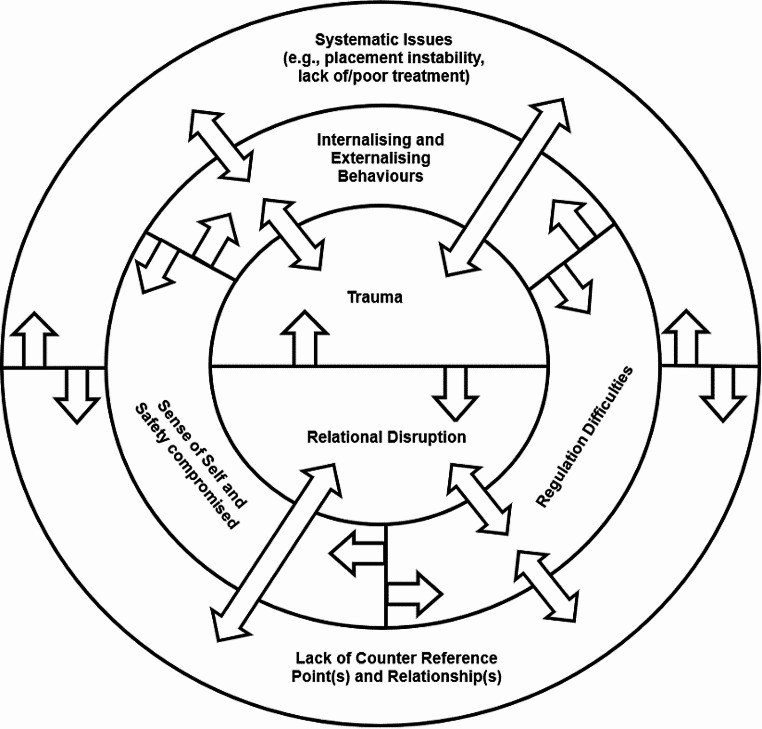
The chaos of complex trauma impacts and constructs (Inner: Precipitating; Middle: Presenting; Outer: Perpetuating)

Agreement about a cycle was present in the groups, yet within the member checking survey, while a majority (7) agreed with the presentation of the cycle, one (1) neither agreed nor disagreed, and two (2) participants disagreed with it. Participants’ critiques of the cycle presented were notably in agreement with conversations between the research team, including:

#### Neither

*It shows as cycle, but it is really more 3D as they get worse or better depending on interventions and changes.*

#### Disagree

*I don’t agree with the cycle process that implies causation or some kind of sequence - so I am not sure that this [is] actually accurate.*

#### Disagree

*The cycle looks very neat and linear, which is not how trauma is experienced. I think it should look more like a mind map or messy, non-linear diagram which shows how experiences, development and relational connections are all interwoven to contribute to what makes the trauma ‘complex’.*

## Discussion

### Non-Linearity (Chaos) of Complex Trauma

The need to adjust to the nonlinearity mentioned for the cycle from member checking aligned with the interpretative analysis and conceptual of the research team as *the chaos of Complex Trauma* noted in *theme three* but were limitations of the visual media. Through further discussions within the research team, a more suitable representation was created as Fig. 5 including three higher-level domains captured to represent the chaos of complex trauma, but this was not member checked:

#### Precipitating Domain

Initial experiences – traumatic events with disrupted relationships and core unsafe and dysfunctional reference points.

#### Presenting domain

The impacts of trauma: internalising and externalising behaviours that show the lack of safety, dysregulation, and disrupted attachment style.

#### Perpetuating Domain

Impacts interact with systematic issues; there is a lack of counter reference points and relationships (e.g., rotational carer in out-of-home care)

This 2D diagram better represents the interconnectedness of each factor at play across the domains, both stemming from and causing complex trauma, this can better be described via analogy.

### Chaotic Bumper Cars

Each domain is a different bumper car colour of variable size, make, and power; each domain has multiple cars representing the various factors/elements. As the cars move, they bump into one another, moving in chaotic motions with variable speeds. As they collide with one another, they cause damage to the other car and themselves but also cause that car to move and bump into another car and another and so on. Any car can bump and influence/damage/move another car. As the cars move, without intervention/change/new pathways for behaviour, they continue to cause damage to one another – i.e., more trauma, more impacts emerge for, and happen to, the child or young person, etc.

The cars only slow or are repaired if a mechanic intervenes on one of them, a car is removed from the track, or a speed limit is implemented to slow the damage down—i.e., stable placement, targeted effective treatment, relational stability, improved capacity. Collision in our lives is normal, but children experiencing the chaos of complex trauma turn the collisions of life into a recursive issue without the patterns and pathways to repair and recuperate.

### Analysing Concept Maturity

A way forward here could be to focus on the presentation of the array of complex trauma impacts and to have a better understanding of how real-world cases are worked on with real people and effective treatment components. However, it is difficult to conceptualise how we would measure effectiveness with this data, given that we cannot communicate concretely what complex trauma is for it to be treated. Throughout the results, consistent references to aspects of concept maturity bring into question the usefulness of complex trauma as it is currently used. Compared with Morse et al. (1996) indices of concept maturity, several concept anatomy and maturity factors were lacking in the findings, discussed below.

#### Definition

There is a stark lack of clarity and several competing definitions between developmental and relational trauma. This blurring is compounded by using the term complex in other conceptual spaces like that of Complex PTSD, associating it with a diagnosable illness. This issue has persisted for some time (see Tarren-Sweeney, 2008). We need to be clearer on what is meant by complex itself and be sure this is a consensual definition with the lived experience population to afford maturity to this concept – recent research has highlighted how the experience can be heterogeneous (Hovane et al., 2023).

#### Characteristics

The characteristics are poorly defined, particularly between cause/experience and effect/impact. The notion of complex trauma refers to the wide array of impacts and the seemingly undefined possible multiple or consistent traumas that cause one to “have complex trauma” (Cook et al., 2005; Wamser-Nanney, 2016).

#### Boundaries

There are no clear boundaries to these characteristics. As users of this term, participants noted their own confusion and lack of clarity and that their examples of what “is not complex trauma” could be complex trauma but with fuzzy boundaries. Potentially the impetus for new disorders (e.g., DTD; van der Kolk, 2005) is to give clear boundaries, but the fit of these disorders for complex trauma from abuse and neglect is still unknown.

#### Preconditions and Outcomes

There are no shared preconditions and outcomes. There is an expectation that the behaviours change over the lifespan but also that there is some ineffable notion of professionals knowing which cases are complex trauma and which are not. The only elements that seem uniform for trauma are that there is an integrity-threatening event, and that dysfunction follows. However, there is even a debate, as seen above, given impacts or distress may present after meaning-making or re-narrating early life trauma through counter reference points (for example, where a child experiences sexual abuse by her father as ‘normal’ – not having any other reference point –changes this view when she learns what sexual behaviour and intimacy is, or sees the interactions of other children with their fathers that are not sexual). This raises a question: is complex trauma valid after that re-narrating of the experience and impacts start to present?

#### An Emerging Concept

According to Morse et al.’s (1996) indices of maturity, complex trauma is an emerging, or even immature, concept for practice, research, and further theorising. Further qualitative enquiry with a larger sample, a further literature review for research clarity or uniformity, akin to Zumstein and Riese’s (2020) analysis of Severe and Persistent Mental Illness, and a synthesis of these is required to understand better what is emerging. Decisions of which characteristics (impacts perhaps) are core to complex trauma, what is a precondition, and what is an outcome is needed on a larger scale, especially for overcoming the question of whether complex trauma is the initial experience, the follow-on effects, or both. Qualitative investigations with further groups can define this richness (thick descriptions) through potential case analysis and feedback on the impacts array (Fig. 1) by professionals and those with self-identified lived experiences of complex trauma from abuse and neglect. Asking professionals to succinctly define complex and non-complex trauma or provide cases for coding may well unify some of these discordant elements.

### Limitations

The paper is best seen as a pilot of ideas and considerations; while this is a strength from a field perspective, it is a limitation of the study itself. We can claim that 10 professionals in the area share these views, but the limited sample restricts the transferability of these findings beyond the child protection, Australian, and professional contexts. This limited sample was gathered through networks of the researchers, which may add an aspect of bias despite the small field on which to draw. The disparate nature of the participant’s areas of work furthers the pilot consideration as both a strength showing that the results are not solely reliant on a homogeneous sample and potentially shared views across fields, but also a limitation as they may represent rare views in their respective fields and were selected non-randomly. Further testing of the acceptability of these ideas, or their alignment with other clinicians’ case descriptions, is needed to improve trustworthiness and transferability through a variety of discipline-specific investigations.

Furthermore, a major product of the discussion was a product of the research team’s interpretation (Chaos model and analogy). This disconnect between the results-analysis proper (interview transcripts and survey) and this output again limits the possible transferability of the findings. Note, however, that the member checking allows trustworthiness for integration of this interpretation (Birt et al., 2016). Also note that any qualitative research analysis, including quote presentation, is never solely a property of participants’ utterances alone but always a hermeneutic interactive interpretation by analysts (Braun & Clark, 2021; Guerin, 2016). Still, further confirmation in a separate and larger sample will improve the conclusions and overcome this limitation.

Finally, the limitations of the analytical frame should be noted. While this piece attempts to convey new conceptual meaning to complex trauma as informed by both author and participant knowledge, the purpose of analysis as a consensus finding and homogenising activity cannot be ignored. Further investigation, especially with lived experience, will almost certainly highlight the need for heterogeneous understandings at all levels of interaction with complex trauma, and this is never something that analytical processes convey well, given the understanding of reliability or repeatability as trustworthiness. Further research should be wary of the same box-like classification that the DSM presents, given the issues participants raised and that classification is more functional in government systems than understanding people’s lives.

## Conclusions

Several things can be suggested concerning our research questions—as points to confirm further with larger samples. First, the impacts and constructs related to complex trauma seem extremely varied and change markedly across the lifespan. As such, providing direct and simple lists of behaviours, symptoms, or impacts is unlikely to be possible. For lack of a better phrase, it is more complex than that.

Second, the impacts did not fit neatly with the current diagnostic criteria, nor are they reflected holistically in any diagnostic tool (Morelli & Villodas, 2022). This may be a trend found beyond complex trauma alone. There were conversations about the need for new diagnostic tools and criteria for complex trauma and its own diagnosis, but also a critique of the need for diagnosis itself as more for funding and treatment structure than explaining these children and young people’s experiences. Remembering that diagnosis can be helpful at times for informing treatment. However, the interaction between funding and diagnosis is specific to the Australian context, with other jurisdictions needing investigation.

Third, these impacts appear as though they could be grouped into symptom clusters/categories but potentially only with a radical shift in how we understand this clustering, especially to stop children and young people with complex trauma from being framed as the source of disordered behaviour. Instead, further investigation should start with trauma-related behaviour being understood as a reasonable reaction to their context/ experiences.

Fourth, complex trauma from child abuse and neglect appears difficult to distinguish from other types of trauma. These experts tended to use different trauma terms interchangeably. The pervasive and interpersonal nature of abuse and harm, as well as the multidimensional effects from child abuse and neglect often occurring at an early age, means other types of trauma were considered complex (e.g., relational and developmental trauma). In contrast, not all complex trauma was said to be developmental or relational. Further definition and agreement checking are needed.

Fifth, like the aetiology of PTSD or acute stress disorder, there is an event or experience of profound harm and threat present within the background of children and young people who develop complex trauma. Unlike these disorders, however, participants suggested that for complex trauma, there is a requirement for prolonged or sustained periods of multiple events and experiences of profound harm, threat, and deprivation and that these be accompanied or occur within the context of a disruption to key attachment relationship/s (with a parent/s or caregiver/s). The failure or absence of an attachment relationship that provides unconditional regard, nurturance, and safety, with repeated threat and harm, were critical factors for complex trauma in this small sample (also see van der Kolk, 2005).

Sixth and finally, we suggest the chaotic cycle of the effects of complex trauma. This cycle could continue throughout the lifespan while changing in behavioural presentation; those behaviours could be of the same type, intensity, or reactions. Systems of welfare and care were spoken about as spaces that perpetuate low-attachment or transient relationships, elements core to participants’ description of complex trauma and its presentation. However, the reality of their experiences and reactions may be lost as they meet different diagnostic criteria as these changes occur. Moreover, continued exposure to the same low-attachment or transient relationships across contexts and systems tends to provide a lack of counter reference points and corrective validating experiences, which, in turn, may function to maintain complex trauma presentations across the lifespan. If this cycle is repeated over time, a child’s externalising and/or internalising behaviours could become so deeply ingrained that they become a way of being, a lens through which they see themselves and the world, and how they relate to others rather than acute distress. When this occurs, personality pathology seems to present as a complex trauma impact.

Altogether, we are confronted with the reality that complex trauma as a concept is extremely broad, non-specific, and overlaps with other concepts. The seeming immaturity of the concept means our next steps are to define and delineate what is meant more widely, including in the literature, further research with professionals who work with complex trauma, but more importantly, hearing the voices and views of those who live with complex trauma – though their possible identification eludes us. We must privilege the input of those with complex trauma above professionals as they bear the consequences of these labels and processes.

## Electronic Supplementary Material

Below is the link to the electronic supplementary material.

Supplementary Material 1

Supplementary Material 2
