# Supplementary material for: Complex Trauma from Child Abuse and Neglect “I’m not Sure We’re even All Talking about the Same Thing and We’re Probably Not”:
Source: J Child Adolesc Trauma. 2024 Aug 3;17(4):1151–68. doi: 10.1007/s40653-024-00648-z (PMC11646253; doi:10.1007/s40653-024-00648-z)
Supplement: Supplementary file 1 — Supplementary Material 1 [file 40653_2024_648_MOESM1_ESM.docx]

**Complex Trauma Focus Group Protocol**

This protocol represents a general guide for use with each of the focus groups. The use of a general protocol guide provides some degree of consistency across the focus group consultations as well as the opportunity for open discussion and reflection. Within the protocol, some detail includes general examples and scripts in italics that can be used by the facilitators.

**Prior to Commencement of the Focus Group**

The facilitators to provide participants with required information in advance (e.g., Participant Information Sheets etc.). The facilitators to send participants an email with a link to an electronic information sheet, consent form, and demographic survey.

**Commencement of the Focus Group**

The facilitators provide participants with a welcome and discuss general housekeeping (e.g., Acknowledgement of Country and victims/survivors, explanation of consent, participation in the study, details of the research project, the aims of the focus group).

***Acknowledgement of Country***

**Adelaide (South Australia).** *We would like to acknowledge the Kaurna people as the custodians of the lands, waterways, and skies of the Adelaide region where I am today. We pay respect to Elders past and present. We also acknowledge and respect the Kaurna people’s cultural, spiritual, physical and emotional connection with their land, waterways, skies and community. We acknowledge that this virtual focus group is taking place on lands of the First Nations People across the country, and we extend our respect to those communities.*

**Perth (Western Australia).** *We would like to acknowledge the Whadjuk people as the custodians of the lands, waterways, and skies of the Perth region where I am today.* *We pay respect to Elders past and present. We also acknowledge and respect the Whadjuk people’s cultural, spiritual, physical and emotional connection with their land, waterways, skies and community. We acknowledge that this virtual focus group is taking place on lands of the First Nations People across the country, and we extend our respect to those communities.*

***Acknowledgement of Victims and Survivors***

*We would like to acknowledge any victims and survivors and their supporters who are present with us today. We also wish to pay our respects to all victims and survivors of all child maltreatment, including abuse and neglect, and acknowledge the enormity of the trauma they suffered as children. [We] endeavour to learn to better protect children and young people. We dedicate our ongoing efforts to improving supports for victims and survivors; preventing child abuse and neglect and ensuring our children and young people are safe from harm.*

***Introductions***

The facilitators introduce themselves to participants (e.g., their professional background and role in the research project). Facilitators to ask participants to give a basic and brief introduction about themselves (e.g., name and service/professional role). The facilitators will clarify whether participants have completed the electronic consent form and demographic survey (i.e., basic information about themselves such as age, professional role). If any participant has not completed this information prior to commencement of the focus group, they will be asked to do so now.

***Introduction to the Research Project***

The facilitators include a basic overview of the research project and collaborative nature of the focus group as well as the main focus of the group.

*We are going to provide you with a rundown of what we are doing today and covering off some housekeeping issues. As we go through the session, we are hoping to get your thoughts and practice wisdom on the topics discussed. We would like these focus groups to bring rich and collaborative discussion where each of you are able to share your experiences, thoughts, and ideas. In this focus group, we will ask you to think critically about the presentation of children with complex trauma from abuse and neglect. We would like to hear your general thoughts, ideas, and reflections on the conceptualisation of trauma-related diagnoses, constructs, and symptoms in children with complex trauma based on your practice experience. In this focus group, we also want to hear about any challenges that you face when it comes to complex trauma and symptoms. We will also be getting your thoughts on the factors that contribute to the development and maintenance of complex trauma (e.g., keeping in mind why some children go on to develop complex trauma and other children don’t). By the end of the focus group, we will hopefully come up with some themes from the session. Then we will close with any final comments and thoughts.*

*We ask that, wherever possible, only one person speaks at a time. In order to make sure that the live transcription function works effortlessly, we will ask that you please login to a device and access Microsoft Teams individually. Even if you belong to the same organisation/workplace, we need you to login as an independent participant rather than sit together at one computer. We are happy for you to leave your video on or off throughout, whatever you prefer, but if you could turn it on when you are speaking that would be great.*

Following introductions to the research project and focus group, the facilitators will provide opportunity for participants to ask any questions or share any concerns and will check that participants provide their consent to proceed with the group.

**Explanation about What Information Will be Collected What it Will be Used For.** *As part of this research project, we will be collating and summarising the discussion we get from this group and other groups. Please note that no comments or details will be attributed to you or your service/agency. As part of the research findings and write-up, we will generally describe how many people participated across the groups and the broad types of services that contributed. There will be no specific or individual attendance information that will be provided. We will be using the recording and live transcription function in Microsoft Teams for the group discussion. This is an automatic process that happens in near real time and no one outside of the research team sees the recording and transcription. Using this function allows us to be present in the discussion with you. Using recording function gives us the chance to check details of the transcripts later so that we can accurately and truthfully represent what the groups tell us and discuss. It’s also useful for anyone that might be hard of hearing as the transcription is available as we go. Alongside the live transcription, we will also be having one of us [reference to other facilitator] here taking notes for the whole of group discussion. Although we will not be sharing the transcripts and notes, we will be removing any identifying information from the transcripts and notes, and these will be stored in a secure location.*

**Participation in the Focus Group.** *We hope that this feels like a safe space and are really keen to hear your thoughts and ideas and for you to share your knowledge and expertise. To keep this feeling like a safe space, we ask that everyone is respectful of each other’s thoughts, opinions, reflections, and experiences. All thoughts and opinions are valid, and there are no right or wrong ideas. There is a fair bit to discuss, so at times, we may need to interrupt in order for us to stay on topic and on track in terms of time. We will ask questions along the way and are hoping that this will prompt some rich discussion and creation of meaning. Please remember that we will be talking about child abuse (e.g., physical abuse, sexual abuse, emotional abuse) and neglect and this may be confronting for some and may trigger uncomfortable emotions. Although we know that you all have experience in this area, if this does occur, please let us know, make sure you take care of yourself, or step out momentarily and we can check in with you later. You do not have to answer any questions that you don't feel comfortable answering and you can stop participating at any time. We can also help you find available services to support you if you need. Lastly, we do not anticipate that this will be an issue with this group, but just to note that if anything came up that made us concerned about a child’s safety and risk, or improper or harmful practice* *then we will be required to report it.*

**Focus Group Discussion and Prompts**

The purpose of the focus group is to facilitate discussion between the participants and encourage them to share their ideas and thoughts and draw on their knowledge and experience. The facilitators will guide the discussion and highlight commonalities and themes as the discussion progresses. The facilitators may take notes and document preliminary themes and codes for analysis. The facilitators will take every effort to ensure their understanding by verbally summarising and reflecting back complex and ambiguous points made by participants.

Examples of general questions to facilitate group discussion:

- *What symptoms and/or constructs do children and young people with a background of child abuse and neglect experience and present with?*
- *How do these symptoms compare with the current diagnostic criteria?*
- *Are these symptoms reflected thoroughly and holistically in any one diagnostic tool?*
- *Are any symptoms missing from the general diagnostic criteria related to trauma?*
- *Can these symptoms be grouped into symptom clusters/categories?*
- *What makes complex trauma from child abuse and neglect distinct from other types of trauma?*
- *How is complex trauma identified and/or recognised from other types of trauma?*
- *What factors influence the development of complex trauma in children and young people who have experienced child abuse and neglect?*
- *What mechanisms serve to maintain complex trauma presentations into adulthood and across the lifespan for some children and young people who have experienced child abuse and neglect?*

Examples of additional prompts to facilitate group discussion:

- *Can you help us understand what you mean?*
- *Can you talk about that more?*
- *Can you give an example?*
- *What would that look like?*
- If a participant becomes distracted or tangential, refine the focus of the discussion (e.g., *Thank you for your comment, we are mindful of time and that we have a lot to get through and discuss today. But we encourage you to bring this up at the end when we have space for some final thoughts and comments).*
- If a participant is dominating the discussion, then provide some redirection (e.g., *Thank you for sharing your thoughts. What do other people think? Let’s have some comments from others).*
- If participants appear to be in disagreement/argument, then aim to support repair and resolution (e.g., *It’s important that we get all of our concerns out on the table, so that we can be confident that we are not missing anything. I wonder if we can explore why we hold such different views about this topic).*

**Closing of the Focus Group**

The facilitators will aim to provide a brief summary of the main points discussed. If there is any time remaining, then participants will be asked if they have any additional thoughts and ideas they would like to share. The facilitators will thank participants for their participation and contributing to the group consultation and discussion. The facilitators will remind the participants about the follow up survey to be sent via email.
