# Supplementary material for: Complex Trauma from Child Abuse and Neglect “I’m not Sure We’re even All Talking about the Same Thing and We’re Probably Not”:
Source: J Child Adolesc Trauma. 2024 Aug 3;17(4):1151–68. doi: 10.1007/s40653-024-00648-z (PMC11646253; doi:10.1007/s40653-024-00648-z)
Supplement: Supplementary file 2 — Supplementary Material 2 [file 40653_2024_648_MOESM2_ESM.docx]

**Complex Trauma Group Discussion**

**Table 1**

*Codebook for the analysis of complex trauma group discussions
(Key = T: Trauma, CT: Complex Trauma, Ind: Inductive Code, Ded: Deductive Code (source named), GDx: Group Discussion number, Passx: End of coding pass organisational change)*

| Codes | Description |
| --- | --- |
| Contextual Factors | Contextual Factors - link to mechanisms that present with CT  Ded (Guerin, 2016 & internal complex trauma content) |
| Care or Welfare Systems | Comments on the care, welfare, or residential systems  Ded (internal complex trauma content) |
| Transient Relationships | Relationship instability  Ded (internal complex trauma content) |
| Intergenerational issues | As defined by the speaker but generally when previous generations are highlighted as the complexity discussed  Deductive (Internal complex trauma content) |
| Other Considerations | Contextual Elements as harm/part of symptoms (e.g., not neglect if its forced)  Ind GD3 |
| Others reactions to the CT | Mentions or requotes of what others do or say about children with CT  Ind GD1 |
| Defining complex trauma | How the participants define complex trauma as different from other types of trauma  Ded (Group Discussion Protocol) |
| Comparison to other traumas | That CT is like something else (other trauma type)  Ind GD1 |
| Types of trauma | Housing code  Ind GD1 |
| Developmental Trauma | Discussion of Developmental Trauma in particular  Inductive Pass1 |
| Relational Trauma | Discussion of Relational Trauma in particular  Ind GD4 |
| Course into Adulthood | Housing code  Ind GD2 |
| Presentation Changes | Title  Ind GD2 |
| The cycle | That CT is C because it is recursive in symptom and effect  Ind GD2 |
| Counter Reference | Mentions of counter reference points  Ind GD2 |
| Absence of positives | Comments that CT is not having the buffer or positive bits to draw on  Ind GD2 |
| Instigation | Housing for comments about things that instigate the trauma or are the trauma experience  Ind Pass1 |
| Abuse | Complex trauma as abuse  Ded (internal complex trauma content) |
| Experience over impact | That the experience is more important than the impact (e.g., trauma could not present with impacts)  Ind GD2 |
| Harm | comments that harm (to the child) is key  Ind GD3 |
| Harm and neglect together | When harm and neglect for CT are not separated contextually  Ind GD3 |
| Neglect | mentions of neglect  Ded (Internal complex trauma content) |
| Separating factors between T and CT | Title  Ind GD1 |
| Age or developmental stage as key | Age or developmental stage as key to CT  Ind GD1 |
| Impact over experience | That the impacts are different  Ind GD2 |
| Instability unpredictability | Effects of instability or unpredictability  Ind GD2 |
| Meaning making | Comments on the meaning of behaviour or experience changing  Ind GD2 |
| More enduring or intense | Title as a key feature of CT  Ind GD1 |
| Multiple X | Descriptions of CT as being about multiple things e.g., abuse  Ded (Internal complex trauma content) |
| Pervasiveness | comment on the singular versus multiple but over time as well elements of CT  Ind GD1 |
| Severity | Comments about severity  Ind GD1 |
| Relationships | Relationships are key to CT compared to other trauma  Ind GD1 |
| Treatment issues or resistance | CT is about difficulty in treatment of the behaviours  Ded (Internal complex trauma content) |
| Symptom or Constructs of CT | Housing code  Ded (Group Discussion Protocol) |
| Difficult to define | Claims that CT isn’t easy to say a list or examples  Ind GD1 |
| Individual variation | Claims of individual variation making generalisation difficult  Ind GD1 |
| DSM Diagnosis | Mentions of disorders or diagnosis  Ded (Group Discussion Protocol) |
| Examples | Examples of behaviours seen  Ind GD1 |
| Symptoms and Constructs of CT | Describing symptoms or behaviours as generalised concepts Housing  Ind GD 1 |
| Absence of 'Basic Skills' | Claims of basic skills  Ind GD1 |
| Avoidance or Suppression or Masking | Behaviour to avoid the trauma impacts/events (hiding issues)  Ind GD4 |
| Biological or Innate Traits | Claims of Brain or Neurological changes (not firsthand)  Ind GD1 |
| Biological Change | Separation of brain claims into full body biological claims  Ind pass1 |
| Innate | Separation to innate traits or attributes (not bio/body or brain)  Ind pass1 |
| Locus of Control | Title  Ind Pass1 |
| Pleasure Drive | Title  Ind Pass1 |
| Resilience | Title  Ind GD2 |
| Survival Mechanism or Instinct | Title  Ind Pass1 |
| Neuro or Brain Issues | Claims about neurology or brain science as the symptom  Ind pass1 |
| Cognitive | Cognitive symptoms  Ind GD3 |
| Beliefs (errors) | Title  Ind GD3 |
| Cognitive Deficit (memory) | General comments on these issues  Ind pass1 |
| Distorted Thinking | Claims about distorted thinking or patterns that are not reality  Ind pass1 |
| Executive Functioning | Claims about issues with executive functioning  Ind pass1 |
| Sensory Processing Issues | Title  Ind GD 1 |
| Developmental Issues | Title  Ind GD1 |
| Age Dependent Behaviour | Comments that the age basis of behaviour is key  Ind GD3 |
| Educational Troubles | Comments about the education issues as a focus  Ind GD2 |
| Externalising and Internalising | Use of these terms  Ind GD2 |
| Absent or Dissociated or Withdrawal | Title  Ind Pass1 |
| Aggression or Explosive | Title  Ind Pass1 |
| Avoidance of Triggers | Title  Ind Pass1 |
| Disinhibition | Break out code for disinhibition points  IND GD3 |
| Harm | Highlighting harm to someone as key  Ded (Group Discussion Protocol) |
| Others | Harm to others  Ded (Group Discussion Protocol) |
| Self | harm to self  Ded (Group Discussion Protocol) |
| People Pleasing | Title  Ind Pass1 |
| Flashbacks | Title (PTSD link)  Ind GD3 |
| Food Behaviour | Title  Ind GD2 |
| Identity or Sense of self | Title  Ind GD 1 |
| Learnt Behaviour | Comments to the learning of behaviours seen as symptoms  Ind GD3 |
| Neglect | Title  Ind GD3 |
| Personality | Title  Ind GD4 |
| Play | Children’s need to play (fun)  Ind GD1 |
| Regulation | Claims of regulation, including co and self-regulation  Ind GD1 |
| Emotion | Emotion, literacy, and control  Ind GD1 |
| Angry | Title  Ind Pass2 |
| Empathy (lack of) | Comments about empathy or lack of  Ind Pass2 |
| Guilt | Title  Ind Pass1 |
| Overwhelming | Mention of emotion as too much in some way  Ind Pass2 |
| Shame | Title  IND GD1 |
| Worthlessness | Title  Ind Pass1 |
| Relational | Claims of relational needs as a key to CT  Ind GD1 |
| Attachment | Describing complex trauma by issues with attachment  Ded (Internal complex trauma content) |
| Risk Averse or Taking | As titled IND GD1 |
| Sense of Safety | comments that relate to the CT sufferers’ sense of safety (irrespective of reality)  Ind GD1 |
| Triggers | Claims of triggers more broadly  Ind GD1 |
| Threats | Title  Ind GD3 |
| Sexual Behaviour | Focus on sexual behaviour  Ind GD2 |
| Sleep issues | Title  Ind GD2 |
| Treatment focused talk | Talk about how they help them rather than what it is Potentially for full coding as "what it is"  Ind GD1 |
| Can it be resolved | comments about if CT can be resolved, treated to cure, etc.  Ind GD1 |
| Hope and change | Comments that there are positive things in all of this  Ind GD1 |
| Paradoxical treatment outcomes | When the clients of treatment can come out "worse" because they break down the avoidance behaviours  Ind GD4 |
| Symptom to diagnosis or construct comparison | Answers related to contrasting between symptoms and diagnosis info  Ded (Group Discussion Protocol) |
| Alternatives | Housing code  Ind GD3 |
| Chronic Maltreatment | Title  Ind GD3 |
| Dimensional | Dimensional model points  Ind GD3 |
| Threat and deprivation | Title  Ind GD3 |
| Transdiagnostic | Title  Ind GD3 |
| Critiques of Diagnosis approach | Critiques of the diagnosis approach  Ded (Group Discussion Protocol) |
| Challenging Frustrating to do | Title  Ind GD3 |
| Contextual other things involved | That the experience behaviour or causes do not happen alone  Ind GD1 |
| Dysfunctional for Others | Diagnosis is what is problems for others  Ind GD3 |
| Harmful punitive stigma | That diagnoses themselves are possibly harmful or punitive  Ind GD1 |
| Ignore reality for rationale | The reality of behaviour and diagnosis is not aligned for behavioural reasons but funding  Ind GD1 |
| Internal External debate | When the issue is located inside versus outside the person with disorder  Ind GD3 |
| Disability/dysfunction focus as the issue | Title  Ind GD3 |
| Misinterpretation | Diagnosis as misinterpreted (e.g. not disorder trauma)  Ind GD2 |
| No one box fits | Diagnoses do not cover all presenting symptoms or behaviours  Ded (Group Discussion Protocol) |
| Not a focus of assistance | that diagnosis or clustering isn’t a real focus of their work  Ind GD2 |
| Not applicable | Diagnoses are not applicable to CT  Ind GD1 |
| Over generalisation | Over generalisation of the issue  Ind GD1 |
| Stop looking at the case | that the reality gets lost to the diagnosis  Ind GD2 |
| Stops new thinking | Comments about how diagnoses limits option or plans for other possible diagnoses or approaches  Ind GD1 |
| System or rationale constraint | Comments about picking diagnosis or construct because it gets them to their rationale e.g., NDIS funding Ind GD1 |
| Variability in approaches | comments that the approach changes what is understood  Ind GD2 |
| Defence of Diagnosis approach | Defence of diagnosis approach  Ded (Group Discussion Protocol) |
| Helpful for professional | It works for them  Ind GD1 |
| Learning | That clusters are good for people learning  Ind GD2 |
| Provides structure | that clusters or diagnosis approaches give structure to look for more  Ind GD2 |
| Rationale for diagnosis | Reasons to keep the diagnosis model that are not alignment based  Ded (Group Discussion Protocol) |
| Background info | diagnoses as background information  Ind GD1 |
| Need for policy or process in workplace | That diagnosis is part of process or policy for workplace  Ded (Group Discussion Protocol) |
